# Sedentary Behavior as a Risk Factor for Liver Fibrosis Development in Patients with Metabolic Dysfunction-Associated Steatotic Liver Disease (MASLD): A Cross-Sectional Study

**DOI:** 10.3390/jcm14217553

**Published:** 2025-10-24

**Authors:** Antonella Bianco, Caterina Bonfiglio, Isabella Franco, Claudia Beatrice Bagnato, Nicola Verrelli, Dolores Stabile, Endrit Shahini

**Affiliations:** 1 Laboratory of Movement and Wellness, National Institute of Gastroenterology IRCCS “Saverio de Bellis”, Castellana Grotte, 70013 Bari, Italy; isabella.franco@irccsdebellis.it (I.F.); claudia.bagnato@irccsdebellis.it (C.B.B.); nicola.verrelli@irccsdebellis.it (N.V.); 2 Data Science, National Institute of Gastroenterology IRCCS “Saverio de Bellis”, Castellana Grotte, 70013 Bari, Italy; catia.bonfiglio@irccsdebellis.it; 3 Core Facility Biobank, National Institute of Gastroenterology IRCCS “Saverio de Bellis”, Castellana Grotte, 70013 Bari, Italy; dolores.stabile@irccsdebellis.it; 4 Gastroenterology Unit, National Institute of Gastroenterology-IRCCS “Saverio de Bellis”, Castellana Grotte, 70013 Bari, Italy; endrit.shahini@irccsdebellis.it

**Keywords:** metabolic dysfunction-associated steatotic liver disease, sedentary behavior, liver stiffness measurement, actigraphy, physical activity

## Abstract

**Background**: Metabolic dysfunction-associated fatty liver disease (MASLD) affects nearly 20% of the Italian population, with an annual economic burden of € 7.94 billion on the National Health Service, largely due to advanced liver disease and metabolic comorbidities. Progressive liver fibrosis remains the strongest predictor of adverse outcomes. Early diagnosis is crucial, as fibrosis is potentially reversible in its early stages. Sedentary behavior (SB) is one of many modifiable risk factors for several chronic diseases; however, most evidence is based on self-reported data. This study investigates the association between objectively measured daily energy expenditure and liver stiffness in adults with MASLD. **Methods**: We conducted a cross-sectional analysis of 104 adults (18–65 years) with obesity and moderate-to-severe steatosis (CAP ≥ 248 dB/m) from the Obesity-AF study. Daily energy expenditure (METs/day) was assessed via 7-day wrist-worn actigraphy. Liver stiffness (LSM, kPa) was measured non-invasively using transient elastography (FibroScan^®^). **Results**: Lower daily energy expenditure was independently associated with higher liver stiffness. Each 1-MET increase correlated with a −1.239 kPa reduction in LSM (95% CI: −2.012, −0.466; *p* = 0.002). Sedentary individuals (≤1.5 METs) exhibited significantly higher LSM (+0.73 kPa, *p* = 0.03) versus non-sedentary peers, even after full adjustment. Findings were robust across sensitivity analyses. **Conclusions**: Our study shows that SB, objectively measured by actigraphy, is independently associated with increased liver stiffness in patients with MASLD and obesity. Reducing sedentary time may represent a simple, low-cost, and scalable strategy to mitigate fibrosis progression. However, given the cross-sectional design and the specificity of our sample, longitudinal studies are needed to confirm the causal role of SB and to evaluate the effectiveness of targeted interventions in broader MASLD populations.

## 1. Introduction

In recent decades, Metabolic Dysfunction–Associated Steatotic Liver Disease (MASLD), formerly known as Non-Alcoholic Fatty Liver Disease (NAFLD), has emerged as one of the leading causes of chronic liver disease worldwide [1]. It is estimated to affect approximately 30% of the global general population and about 20% of the Italian population, with an annual economic burden of €7.94 billion on the National Health Service, largely attributable to advanced liver disease and metabolic comorbidities [2]. MASLD is defined as ectopic fat accumulation in hepatic tissue in the absence of significant alcohol consumption, and it is strongly linked to several metabolic risk factors such as obesity, type 2 diabetes mellitus, and metabolic syndrome [1]. Observational studies show that patients with MASLD have a higher incidence of cardiovascular disease, a greater disease burden, and increased mortality. Although some analyses suggest that MASLD may act as an independent cardiovascular risk factor even after adjustment for conventional risk factors, its specific contribution is difficult to isolate in clinical practice due to its close interconnection with underlying metabolic dysfunction [3].

Although MASLD is typically asymptomatic in its early stages, it can progress to more serious forms, such as metabolic-associated steatohepatitis (MASH), liver fibrosis, cirrhosis, and hepatocellular carcinoma (HCC) [4]. According to epidemiological studies, 10% to 30% of patients with MASLD develop liver fibrosis, increasing the risk of hepatic complications and mortality. In this context, the degree of liver fibrosis is the most important prognostic factor [5], as it is strongly associated with adverse clinical outcomes such as liver-related mortality [6]. Fibrosis progression accelerates the onset of two critical complications of chronic liver disease: impaired hepatocyte cell function and portal hypertension. Moreover, liver fibrosis has been associated with an elevated risk of cardiovascular events, ischemic stroke, and other metabolic complications [7]. Thus, early detection and timely management of liver fibrosis are crucial for slowing or preventing its progression.

Weight loss of ≥10% combined with a structured physical activity program has been shown to significantly reduce liver fibrosis [8]. Currently, liver fibrosis is assessed using both invasive techniques, such as liver biopsy, and non-invasive methods, including serological tests and advanced imaging modalities like liver elastography. While a therapeutic paradigm shift is underway for MASH with advanced fibrosis, aided by the recent approval of pharmacological agents such as Rezdiffra (resmetirom), a once-daily oral thyroid hormone receptor-β (THR-β) agonist that reduces steatosis, inflammation, and fibrosis by targeting hepatic fat metabolism [9], and Wegovy (semaglutide), a glucagon-like peptide-1 (GLP-1) receptor agonist that addresses both liver pathology and underlying metabolic drivers like obesity and insulin resistance [10], no drug has yet been approved for MASLD in general. As a result, lifestyle changes and behavioral interventions remain the foundation of clinical management. The efficacy of these interventions, however, is dependent on early identification of at-risk individuals and the timely implementation of strategies to halt disease progression before advanced fibrosis occurs.

Prolonged sedentary behavior (SB) is becoming increasingly recognized as a potentially modifiable factor in the pathogenesis and progression of liver fibrosis. SB is recognized as “any waking behavior characterized by an energy expenditure ≤1.5 metabolic equivalents (METs), while sitting, lying, or reclining” [11].

It is critical to differentiate SB from physical inactivity. While physical inactivity is defined as a lack of moderate-to-vigorous physical activity, SB is distinguished by specific postures associated with extremely low energy [12]. Although the physiological effects of SB may overlap with those of physical inactivity [11], SB has negative health consequences regardless of overall levels of physical activity. This suggests that even individuals who meet recommended physical activity guidelines may be more susceptible to metabolic and hepatic dysfunction if they engage in sedentary activities for extended periods of time. Sedentary postures cause a series of multisystemic changes that can persist even in physically active individuals [13]. Insulin resistance develops at both metabolic and musculoskeletal levels and is accompanied by alterations in substrate utilization, characterized by a preferential shift toward carbohydrate oxidation over fat oxidation. Muscular changes include the conversion of muscle fibers from oxidative to glycolytic, which is associated with a gradual loss of muscle strength and bone mass. Muscle inactivity specifically reduces mitochondrial efficiency and number. This metabolic dysfunction has systemic effects, increasing whole-body oxidative stress, including the liver, which can harm liver cells and trigger a fibrotic response [14]. Cardiovascular alterations are characterized by significant deterioration of vascular function and decreased cardiorespiratory capacity. Body composition and metabolic changes include an increase in total fat mass, particularly visceral fat, elevated blood lipid concentrations, and the activation of a systemic pro-inflammatory state [14], as well as a chronic low-grade inflammation in the liver (steatohepatitis) that precedes fibrosis [14]. These physiological changes show that SB is a distinct and independent risk factor that can have a negative and generalized impact on health beyond adherence to physical activity recommendations [14]. Furthermore, epidemiological studies have shown a link between prolonged SB and various pathological conditions, including obesity, insulin resistance, type 2 diabetes, metabolic syndrome, cardiovascular diseases, malignancies, and all-cause mortality [15,16,17,18]. These findings indicate that regular physical activity may not fully mitigate the risks associated with prolonged sedentary behaviour [19].

Given the profound and multifaceted physiological consequences associated with this lifestyle pattern, accurate assessment of this risk factor has become increasingly important in clinical and research settings. However, evaluating SB poses significant methodological challenges, primarily due to the scarcity of validated assessment tools [20]. As a result, the majority of current research relies on self-administered questionnaires with surrogate measures, such as time spent watching television [21]. However, these self-report instruments fall short of fully capturing the complexities of SB, particularly in terms of brief periods of inactivity or nuanced daily patterns. To address these limitations, researchers are increasingly using objective measurement techniques, such as accelerometers, to quantify SB. While some studies have proposed a specific step-based threshold (e.g., 5000 steps per day) to define SB using pedometers, these approaches have significant limitations because they do not distinguish between sitting and standing time or adequately describe inactivity patterns throughout the day [22].

Consequently, accelerometer-based activity monitors have become the preferred method for evaluating SB. Research using these objective measures has found strong correlations between objectively measured SB and health outcomes [23,24]. Furthermore, these technological devices allow continuous and accurate measurement of time spent in physical activity and sedentary states, resulting in reliable and reproducible quantitative data. While objective measures have been used in a few studies to assess physical activity in NAFLD patients [25,26], we are aware of no studies that have specifically focused on individuals with MASLD.

Based on the evidence outlined above, it is plausible to hypothesize that prolonged periods of SB may promote the development and progression of liver fibrosis, even in individuals who engage in overall sufficient levels of physical activity. Accordingly, this observational study aimed to examine the association between objectively measured average daily energy expenditure, expressed in metabolic equivalents (METs) and assessed via wrist-worn actigraphy (ActiGraph GT9X), and liver stiffness, used as a surrogate marker of fibrosis, in a cohort of patients with MASLD.

## 2. Materials and Methods

### 2.1. Participants

The Obesity-AF study (ClinicalTrials.gov identifier: NCT06186869) began in October 2023 at the National Institute of Gastroenterology “S. de Bellis” in Castellana Grotte (Bari, Italy), and is currently ongoing. All participants provided written informed consent before enrollment, and the study followed the principles of the Declaration of Helsinki. It was approved by the local Ethics Committee (De Bellis Protocol No. 1253/CE, 7 June 2023). The data for this analysis were collected between October 2023 and October 2024 and are based on baseline measurements.

The inclusion criteria for the study were as follows: (1) Body Mass Index (BMI) ≥ 30 kg/m^2^ or waist circumference > 94 cm in men and >80 cm in women; (2) age between 18 and 65 years; (3) presence of moderate to severe liver steatosis, diagnosed by FibroScan^®^ (Echosens, Paris, France) with a controlled attenuation parameter (CAP) score of ≥248 dB/m; and (4) alcohol consumption less than 20–30 g/day.

### 2.2. Data Collection

Data were gathered by trained personnel using standardized procedures and questionnaires. Sociodemographic, anthropometric, nutritional, medical, and lifestyle-related information was obtained. Anthropometric measurements, such as body weight, height, and waist circumference, were taken using standard protocols. Body weight and height were measured with SECA devices (Model 700 and Model 206; SECA, Hamburg, Germany), and the maximum measurable height was 220 cm.

Liver steatosis was measured using the CAP score obtained with FibroScan^®^. The stage of liver fibrosis was determined by measuring liver stiffness in kilopascals (kPa) using FibroScan^®^.

Fasting venous blood samples were drawn in the morning using K-EDTA anticoagulant tubes. The following biomarkers were assessed using standard laboratory methods: glycated hemoglobin (HbA1c), fasting glucose, fasting insulin, aspartate aminotransferase (AST), alanine aminotransferase (ALT), gamma-glutamyl transferase (GGT), total cholesterol (TC), high-density lipoprotein cholesterol (HDL-C), triglycerides (TG), and homeostasis model assessment of insulin resistance (HOMA-IR).

Physical activity levels were assessed using the International Physical Activity Questionnaire—Short Form (IPAQ-SF) [27]. Participants wore ActiGraph GT9X accelerometers for seven days to objectively monitor their physical activity and SB.

### 2.3. Outcome Assessment: Measurement of Liver Stiffness (LSM) Using FibroScan^®^

FibroScan^®^, a non-invasive medical device based on transient elastography, was used to measure liver stiffness in real time. Liver stiffness values were expressed in kilopascals (kPa), which are the standard unit of measurement for this technology. An experienced operator performed the FibroScan^®^ technique using standardized guidelines to ensure measurement quality and reliability. Liver stiffness is widely recognized as a reliable and reproducible marker for noninvasive assessment of fibrosis.

### 2.4. Exposure Assessment: Measuring SB Using an ActiGraph GT9X

SB was measured using an ActiGraph GT9X Link LLC, Pensacola, FL, USA, an objective and reliable tool for measuring physical activity and SB. The Actigraph, a wrist-worn portable device, uses a triaxial accelerometer to detect movement. Participants wore the device for seven days straight, and daily activity levels were recorded. The data were processed using validated algorithms to convert activity counts into metabolic equivalent units (METs). SB was defined as time spent in activities with a metabolic intensity of less than 1.5 MET. Each participant’s weekly average METs were calculated and used to represent overall SB exposure.

The Supplementary Materials describe the device’s model and functionality.

### 2.5. Statistical Methods

We analyzed baseline variables using mean ± Standard Deviation (SD) for continuous variables. To compare continuous variables, we used the Wilcoxon rank sum test, while categorical variables were evaluated using the χ2 test. Statistical significance was determined using the 95% Confidence Intervals (CI) for *p*-values of 0.05 or less.

The correlation coefficient (with relative *p*-value) between liver stiffness measurement (LSM) and MET was calculated and plotted in a linear prediction plot [28]. A scatter plot was employed to display the distribution of LSM and METs values.

Regression analysis was used to investigate the relationship between LSM and MET.

Because LSM did not follow a normal distribution, which is required for analysis validity, it was transformed with the natural logarithm. All analysis results are reported as natural logarithms and e^β^.

Six regression models were created. In models *a*, *b*, and *c*, the METs variable was treated as a continuous variable, while in models *d*, *e*, and *f*, the exposure variable was categorised as Not Sedentary (>1.5 METs) or Sedentary (≤1.5 METs).

Models *a* and *d* are univariate models; models *b* and *e* are adjusted for sex and age (<50 vs. ≥50 years); models *c* and *f* are adjusted for sex (female vs. male), age (<50 vs. ≥50), Job status, Kcal Day, Fat Mass, HOMA, and RBC.

The goodness-of-fit indicators of the model (R^2^) were reported to monitor the explanatory power of each model shown.

Initially, confounding variables were chosen from the available literature. The minimum absolute reduction and selection (LASSO) was then used to reduce the number of candidate predictors and choose those that would be most useful for model construction [29].

The variance inflation factor (VIF) was also calculated to rule out multicollinearity, and confounders with a VIF greater than five were excluded [30].

The statistical analysis was performed using Stata statistical software version 19.0 (StataCorp 2025, 4905 Lakeway Drive, College Station, TX 77845, USA).

## 3. Results

One hundred and four subjects with MASLD (mean age 48.6 ± 9.8) were included in the study. Table 1 summarizes the baseline characteristics of participants stratified by measured metabolic equivalents (METs). Eighty-seven participants (83.7%) were classified as non-sedentary (MET greater than 1.5), and 17 participants (16.3%) as sedentary (MET less than or equal to 1.5). Statistically significant differences were observed between these groups for multiple clinical and demographic variables.

Sedentary subjects exhibited significantly higher LSM values, with a mean of 7.56 ± 3.59 kPa, compared to 5.43 ± 1.52 kPa in the non-sedentary group, representing an absolute difference of 2.13 kPa (*p* < 0.001). These findings indicate a greater presence of hepatic fibrosis among individuals with increased SB. CAP values showed no significant differences between the groups, with means of (307.65 ± 33.44 dB/m vs. 303.06 ± 37.42 dB/m, respectively).

A comparison of demographic characteristics revealed no significant differences in age or BMI between categories. The sedentary group had a higher proportion of males (71% vs. 45%, *p* = 0.052), as well as significant differences in haemoglobin (16.60 ± 7.69 g/L vs. 14.31 ± 1.37 g/L, *p* = 0.010) and HDL cholesterol (43.94 ± 8.91 mg/dL vs. 50.61 ± 12.51 mg/dL, *p* = 0.039).

Data on physical activity behaviors supported the group categorization. Sedentary participants spent more hours per day in sedentary activities (11.31 ± 1.85 vs. 9.38 ± 1.14 h, *p* < 0.001) and less time in moderate-to-vigorous physical activity (1.25 ± 0.28 vs. 3.12 ± 1.08 h, *p* < 0.001). Additionally, the sedentary group demonstrated significantly lower daily caloric consumption compared to the non-sedentary group (1749.42 ± 425.45 vs. 2557.89 ± 652.55 kcal/day, *p* < 0.001).

Although not statistically significant, the group with MET ≤ 1.5 had a higher proportion of sedentary workers (94% vs. 76%).

**Figure 1:**
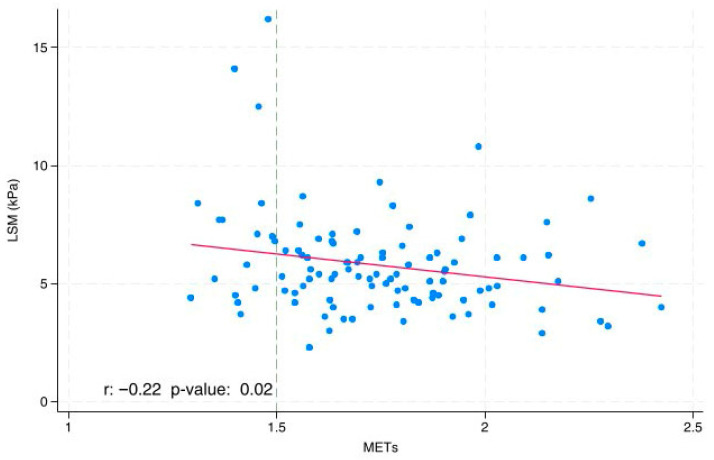
Linear and Scatter Plot between LSM and METs. LSM: Liver stiffness; MET: Metabolic Equivalent Units. r: correlation coefficient.

The line graph (Figure 1) demonstrates an inverse proportional relationship between LSM and METs, with a correlation coefficient (r) of −0.22 and a *p*-value of 0.02. Additionally, a scatter plot was employed to display the distribution of LSM and METs values. Each point on the plot represents the corresponding LSM and METs values for an individual data observation.

Table 2 displays the results of three regression analysis models used to illustrate the relationships between MET and LSM in MASLD patients. Model *a* showed a statistically significant association with METs (β = −0.286; *p*-value = 0.027). In other words, for every one unit increase in MET, the degree of LSM is expected to decrease by 0.777 kPa (e^0.286^).

Because model *a* is univariate, the beta regression coefficient, which estimates the mean decrease in LSM, is solely dependent on the effect of METs, leaving out all potential confounding variables.

Adjusting for gender and age in model b results in a nearly identical reduction in LSM (β = −0.280; *p*-value = 0.038). This indicates that age and gender would have little influence on the decrease in LSM.

In the multivariate model c, the estimated effect of METs on LSM (represented by the β coefficient) was −1.239, with a *p*-value of 0.001.

In other words, for every one-unit increase in METs, the degree of LSM will decrease by −3.377 kPa, controlling for Gender (female vs. male), Age (<50 vs. ≥50), Job, Daily Kcal, Fat Mass, HOMA-IR, and RBC. As a result, the degree of fibrosis is significantly reduced when compared to the previous a and b regression models, which could be attributed to the inclusion of Job, Daily Kcal, Fat Mass, HOMA-IR, and RBC variables (see Table 2).

Table 3 shows regression analyses where the exposure variable was classified as Not Sedentary (METs > 1.5) or Sedentary (METs ≤ 1.5). In model *d*, there was a significant correlation (β = −0.277, *p*-value < 0.001 and 95% CI 0.115–0.459) between the two MET categories and severity of fibrosis. Sedentary behavior is associated with a 0.753 kPa (e^0.277^) increase in LSM, regardless of other factors.

Controlling for gender and age, as in model *e*, the increase in LSM is relatively constant. The coefficient value is β = −0.286 (*p* = 0.001, 95% CI 0.118 to 0.454), which equals 0.777 kPa (e^0.286^). This suggests that age and gender have a minor impact on the rise in LSM (Table 3).

In the multivariate model *f*, the estimated effect of sedentariness versus inactivity on LSM (represented by the β coefficient) was 0.269 with a *p*-value of 0.009, corresponding to 0.731 kPa (e^0.269^). This lower estimate compared to the previous *d* and *e* models could be attributed to the inclusion of Work, Daily Kcal, Fat Mass, HOMA, and RBC variables.

## 4. Discussion

MASLD is now one of the main challenges facing global healthcare systems, not only because of its high prevalence, estimated at more than 11.6 million people, but also because of its multisystemic impact and the significant economic burden it imposes [2]. If MASLD is not diagnosed and treated early, it can progress to advanced stages [2]. It is linked to an increased risk of numerous other diseases [31]. The risk of adverse hepatic events increases exponentially with fibrosis stage, highlighting fibrosis’s central prognostic role [32].

Our findings show that higher SB is significantly associated with increased LSM, implying a higher risk of fibrosis development or progression in subjects with MASLD. In contrast, higher levels of physical activity are associated with lower LSM values, indicating a protective effect against hepatic fibrosis. Higher average daily METs correlate significantly with lower LSM measurements.

This correlation is supported by linear regression analysis, which is rigorously corrected for major confounding variables such as gender, age, occupation, daily calorie intake, fat mass, HOMA-IR, and red blood cell count. Each one-unit increase in MET resulted in a substantial and statistically significant decrease in LSM. Sedentary subjects (≤1.5 MET) had significantly higher LSM values than non-sedentary subjects (>1.5 MET). These findings indicate that low daily energy expenditure is a modifiable determinant of fibrotic progression, independent of other metabolic factors.

Our findings support the notion that SB, in addition to its well-documented metabolic and cardiovascular consequences [14,33,34,35], is significantly associated with MASLD and progression to more severe conditions [36]. Excessive and prolonged SB may cause insulin resistance, vascular dysfunction, a shift in substrate utilization toward carbohydrate oxidation, a shift in muscle fibers from oxidative to glycolytic type, reduced cardiorespiratory fitness, loss of muscle mass, strength, and bone mass, and increased total body fat mass, visceral fat deposition, blood lipid concentrations, and inflammation [14]. It is important to emphasize that the association between SB and MASLD is particularly pronounced in individuals with moderate-to-severe hepatic steatosis [36]. Recent studies suggest that this association loses statistical significance in populations with milder steatosis (e.g., lower CAP values), likely because advanced steatosis reflects a more severe metabolic dysfunction that is especially sensitive to lifestyle-related factors such as SB [37,38].

The physiological plausibility of our results is corroborated by extensive literature demonstrating the negative multisystemic effects of prolonged SB, regardless of overall physical activity levels [14,15]. Chronic low energy expenditure can promote hepatic fibrogenesis via a variety of mechanisms. Prolonged sitting can cause mild chronic inflammation, as evidenced by elevated circulating cytokines and C-reactive protein [14], both of which are known triggers of hepatic stellate cell activation, the key cellular event in the development of fibrosis. In addition, SB is linked to insulin resistance and dyslipidemia [14], metabolic changes that exacerbate hepatic lipid accumulation and lipotoxicity, resulting in a pro-fibrotic hepatic environment. In our study, the reduction in LSM observed with higher METs suggests that increasing daily energy expenditure, even through light-intensity activities, may have a positive impact on these pathways, potentially slowing liver fibrotic progression.

In Italy, MASLD costs the National Health Service around €7.9 billion per year, or 6% of total healthcare expenditure [2]. Over 60% of this is due to metabolic complications, specifically diabetes mellitus, while advanced liver complications (e.g., cirrhosis, HCC, transplantation) cost more than €1 billion. This disease also causes over 13,400 additional deaths each year, with hepatic complications being the leading cause of death [2]. In this context, interventions that can reduce MASLD and slow the progression of fibrosis are beneficial not only clinically but also in terms of healthcare and economics.

Because there are no approved drug therapies for MASLD, lifestyle interventions remain the main strategy. Our study shows that increasing daily energy expenditure, even through light-intensity activities, can positively influence the metabolic and inflammatory pathways involved in fibrogenesis. According to World Health Organization (WHO) recommendations [39], replacing sedentary time with physical activity of any intensity, even light activity, improves metabolic and liver health. The WHO recommends limiting sedentary time and frequently interrupting long periods of SB, particularly at work or home. Effective strategies include taking active breaks every 30–60 min (e.g., 2–5 min walks), utilizing standing or dynamic desks, setting digital reminders to move, and holding structured walking meetings at work [39]. Furthermore, health, school, and community awareness campaigns can help to shift toward more active lifestyles.

The study has both strengths and some limitations. One of the key strengths of our study is the objective measurement of the exposure variable. While many studies use self-reported questionnaires that are prone to recall bias and social desirability bias, actigraphy provides continuous, accurate, and unbiased data on energy expenditure patterns over time. This methodological rigor improves the validity of the observed association. Additionally, the use of liver stiffness measurement (FibroScan) as an outcome variable enables a noninvasive, quantitative, and reproducible assessment of liver fibrosis stage, thereby overcoming some of the limitations of serum biomarkers or clinical scores.

However, the cross-sectional design of the study precludes the establishment of a causal relationship determining the temporal direction of the association observed between SB and increased liver stiffness. In particular, we cannot rule out the equally plausible alternative hypothesis that more advanced liver fibrosis may reduce functional capacity, exercise tolerance, and motivation to engage in physical activity, thereby promoting SB.

While actigraphy is a reliable and objective method for assessing physical activity and SB, it does not differentiate between body postures (e.g., sitting versus standing) or the specific types of low-energy activities, which may influence metabolic outcomes. Furthermore, the cohort studied was selected using very specific criteria: all participants were obese and had moderate-to-severe hepatic steatosis. Consequently, our findings cannot be confidently extended to non-obese MASLD patients, those with mild steatosis, or the general population with metabolic dysfunction. This limitation is particularly relevant at a time when MASLD is affecting a growing number of individuals who are of normal weight or have a “lean” metabolic phenotype.

The potential influence of unmeasured or residual confounding factors, such as detailed dietary patterns, sleep quality, or psychosocial stressors, cannot be ruled out.

Another critical issue concerns the low prevalence of subjects classified as sedentary (16.3%), which is significantly lower than the epidemiological estimates reported in other cohorts of obese patients. This discrepancy suggests a possible selection bias: it is plausible that individuals with highly sedentary behavior are less likely to participate in clinical studies, especially if they require prolonged use of wearable devices or repeated instrumental assessments. This underrepresentation may have attenuated the observed association and limited the ability to explore fibrotic risk in more sedentary individuals robustly.

Therefore, future longitudinal and interventional studies with larger, more diverse samples are warranted to confirm these findings and to further explore the Dose–Response relationship between SB and liver fibrosis progression in MASLD.

## 5. Conclusions

Our findings show a link between low daily energy expenditure and increased liver stiffness in MASLD patients. These results highlight the importance of incorporating SB reduction in addition to structured exercise as a key component of non-pharmacological strategies for slowing the progression of liver fibrosis. While new FDA-approved drug therapies for steatohepatitis with significant liver fibrosis are being developed, simple, low-cost, and easily implementable lifestyle interventions (e.g., regularly interrupting sedentary time and increasing light daily activity) remain critical for liver prognosis and the disease’s economic burden. Future longitudinal and interventional studies are needed to confirm these findings and assess the effectiveness of programs aimed at reducing SB in slowing the progression of liver fibrosis in the early disease stages.

## Figures and Tables

**Table 1:** Characteristics of the sample are divided into sedentary (≤1.5 METs) and non-sedentary behavior (>1.5 METs).

	All Sample *	Non-Sedentary	Sedentary	*p*-Value ^¥^
N	104	87	17	
LSM (kPa)	5.78 (2.13)	5.43 (1.52)	7.56 (3.59)	<0.001
CAP (dB/m)	303.81 (36.69)	303.06 (37.42)	307.65 (33.44)	0.64
Age (years)	48.6 (9.8)	49.2 (9.2)	45.2 (12.2)	0.11
Gender (%)				
Female	53 (51.0)	48 (55)	5 (29)	0.052
Male	51 (49.0)	39 (45)	12 (71)	
BMI (kg/m^2^)	35.56 (4.16)	35.43 (4.08)	36.20 (4.66)	0.49
Fat Mass (Kg)	52.12 (16.82)	50.86 (15.68)	58.57 (21.20)	0.094
Fat Free Mass (kg)	31.27 (11.64)	31.06 (11.18)	32.36 (14.15)	0.68
Glucose (mg/dL)	98.00 (11.51)	98.07 (12.25)	97.59 (6.74)	0.87
AST (U/L)	21.29 (6.83)	20.98 (6.34)	22.88 (8.99)	0.30
ALT (U/L)	28.03 (14.55)	27.14 (13.79)	32.56 (17.73)	0.16
GGT (U/L)	29.80 (21.23)	29.36 (21.67)	32.06 (19.28)	0.63
HDL (mg/dL)	49.52 (12.21)	50.61 (12.51)	43.94 (8.91)	0.039
TC (mg/dL)	199.65 (39.91)	201.04 (38.07)	192.56 (48.95)	0.43
TG (mg/dL)	137.62 (74.30)	132.06 (70.47)	166.07 (88.40)	0.084
HbA1c (%)	5.50 (0.38)	5.51 (0.38)	5.48 (0.39)	0.81
HOMA IR	4.66 (2.17)	4.57 (2.13)	5.13 (2.37)	0.33
WBC (10^3^/μL)	6.33 (1.46)	6.30 (1.47)	6.50 (1.44)	0.62
RBC (10^6^/μL)	4.97 (0.46)	4.95 (0.45)	5.07 (0.53)	0.34
Haemoglobin (g/L)	14.69 (3.39)	14.31 (1.37)	16.60 (7.69)	0.010
Sedentary (h/day)	9.70 (1.46)	9.38 (1.14)	11.31 (1.85)	<0.001
Total MVPA (h/day)	2.81 (1.21)	3.12 (1.08)	1.25 (0.28)	<0.001
Kilocalories ^Ϯ^ (day)	2420.45 (689.28)	2557.89 (652.55)	1749.42 (425.45)	<0.001
Smoker (%)				
Never/Former	90 (86.5)	75 (86)	15 (88)	0.82
Current	14 (13.5)	12 (14)	2 (12)	
Job type (%)				
Not sedentary	22 (21.2)	21 (24)	1 (6)	0.092
Sedentary	82 (78.8)	66 (76)	16 (94)	
Work (%)				
Managers and Professionals	18 (17.3)	14 (16)	4 (24)	0.68
Craft, Agricultural, and Sales Workers	57 (54.8)	47 (54)	10 (59)	
Elementary Occupations	8 (7.7)	8 (9)	0 (0)	
Housewife	5 (4.8)	5 (6)	0 (0)	
Pensioner	5 (4.8)	4 (5)	1 (6)	
Unemployed	11 (10.6)	9 (10)	2 (12)	
Marital Status (%)				
Single	26 (25.0)	20 (23)	6 (35)	0.46
Married or living together	69 (66.3)	60 (69)	9 (53)	
Separated or divorced	7 (6.7)	5 (6)	2 (12)	
Widow/er	2 (1.9)	2 (2)	0 (0)	
Education (%)				
Primary school	1 (1.0)	1 (1)	0 (0)	0.24
Secondary school	15 (14.4)	14 (16)	1 (6)	
High School	59 (56.7)	51 (59)	8 (47)	
Graduate	29 (27.9)	21 (24)	8 (47)	

^¥^ Continuous variables were compared using the Wilcoxon rank-sum, while the Chi2 test was used for categorical variables. * Mean (SD). ^Ϯ^ Kilocalories consumed, calculated using ActiGraph@. MET: Metabolic Equivalent Units; LSM: Liver Stiffness; CAP: Controlled Attenuation Parameter FibroScan^®^; BMI: Body Mass Index; AST: Aspartate Transaminase ALT: Alanine Amino Transferase; GGT: Gamma Glutamyl Transferase; HDL: High-Density Lipoprotein; TC: Total Cholesterol; TG: Triglycerides; HbA1c: Glycated Hemoglobin; HOMA-IR: Homeostasis model assessment for insulin resistance; WBC: White Blood Cells; RBC: Red Blood Cells; MVPA: Moderate and Vigorous Physical Activity.

**Table 2:** The regression model’s expected LSM values (in natural logarithms and e^β^), with the METs exposure variable expressed continuously.

LSM (kPa)	β	e^β^	*p*-Value	95% CI
Model *a*				
R^2^ 0.0477				
METs	−0.286	−0.777	0.027	−0.539; −0.033
Model *b*				
R^2^ 0.0488				
METs	−0.280	−0.761	0.038	−0.544; −0.016
Model *c*				
R^2^ 0.2457				
METs	−1.239	−3.377	0.001	−1.923; −0.554

Model *a*: Univariate model; Model *b*: adjusted for Gender and Age (<50 vs. ≥50 years), Model *c*: Gender (Female vs. Male), Age (<50 vs. ≥50 years), Job, Kcal Day, Fat Mass, HOMA-IR, RBC. β and the 95% CI are expressed in natural logarithms. HOMA-IR: Homeostasis model assessment for insulin resistance; RBC: Red Blood Cells. METs: Metabolic Equivalent Units. β: coefficient regression.

**Table 3:** The regression model with expected LSM values (expressed in natural logarithms and e^β^) and a categorical MET exposure variable (not sedentary vs. sedentary behavior).

LSM (kPa)	β	e^β^	*p*-Value	95% CI
Model *d*				
R^2^ 0.1016				
METs >1.5 vs. ≤1.5	0.277	0.753	0.001	0.115; 0.459
Model *e*				
R^2^ 0.1064				
METs > 1.5 vs. ≤1.5	0.286	0.777	0.001	0.113; 0.447
Model *f*				
R^2^ 0.1986				
METs > 1.5 vs. ≤1.5	0.269	0.731	0.009	0.069; 0.468

Model *d*: univariate. Model *e*: adj for gender (Female vs. Male) and Age, Model *f*: adj for Gender (Female vs. Male), Age (<50 vs. ≥50), Job, Kcal Day, Fat Mass, HOMA-IR, RBC. METs > 1.5: Not sedentary; METs ≤ 1.5: Sedentary. β and the 95% CI are expressed in natural logarithms. HOMA-IR: Homeostasis model assessment for insulin resistance; RBC: Red Blood Cells. METs: Metabolic Equivalent Units. β: coefficient regression.

## Data Availability

The data presented in this study are openly available at https://doi.org/10.6084/m9.figshare.30021844.

## Supplementary Materials

The following supporting information can be downloaded at: https://www.mdpi.com/article/10.3390/jcm14217553/s1. The device’s model and functionality.

## Abbreviations

The following abbreviations are used in this manuscript:

ALT	Alanine Aminotransferase
AST	Aspartate Aminotransferase
BMI	Body Mass Index
CAP	Controlled Attenuation Parameter
GGT	Gamma-Glutamyl Transferase
HbA1c	Glycated Hemoglobin
HCC	Hepatocellular Carcinoma
HDL-C	High-Density Lipoprotein Cholesterol
HOMA-IR	Homeostatic Model Assessment of Insulin Resistance
IPAQ-SF	International Physical Activity Questionnaire-Short Form
LSM	Liver Stiffness Measurement
MASLD	Metabolic Dysfunction-Associated Steatotic Liver Disease
MASH	Metabolic Dysfunction-Associated Steatohepatitis
MET	Metabolic Equivalent of Task
NAFLD	Non-Alcoholic Fatty Liver Disease
SB	Sedentary Behavior
T2DM	Type 2 Diabetes Mellitus
TC	Total Cholesterol
TG	Triglycerides
WHO	World Health Organization
