# Supplementary material for: Sedentary Behavior as a Risk Factor for Liver Fibrosis Development in Patients with Metabolic Dysfunction-Associated Steatotic Liver Disease (MASLD): A Cross-Sectional Study
Source: J Clin Med. 2025 Oct 24;14(21):7553. doi: 10.3390/jcm14217553 (PMC12609606; doi:10.3390/jcm14217553)
Supplement: Supplementary file 1 [file jcm-14-07553-s001.zip › jcm-3935472-supplementary.pdf]

User Guide

ActiGraph GT9X Link + ActiLife

Activity Monitor: ActiGraph GT9X Link | E.200.6001 | Revision: 6 | Released: 02/18/2020

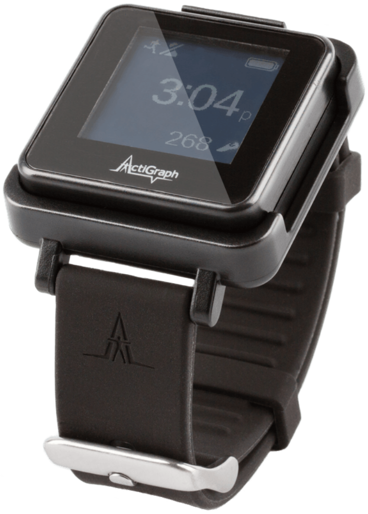

ActiGraph GT9X Link + ActiLife

Activity Monitor: ActiGraph GT9X Link | E.200.6001 | Revision: 6 | Released: 02/18/2020

Contents

Overview.......... 2
What You'll Need.......... 3
Getting Started.......... 4
Setup & Initialization.......... 6
Deployment & Information for Subjects.......... 9
Downloading the Data.......... 12
Viewing the IMU Data.......... 14
Technical Details.......... 15
Frequently Asked Questions.......... 17

Overview

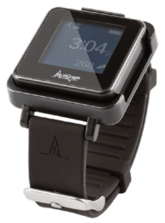

ActiGraph GT9X Link

The ActiGraph GT9X Link is the most sophisticated activity monitor available from the global leader in actigraphy measurement. The ActiGraph Link combines our extensively validated accelerometry measurement technology with a variety of advanced new features, including:

Bluetooth® LE

Enables wireless device features (heart rate monitoring, proximity detection) and communication with ActiGraph mobile applications.

Programmable display

An LCD window displays date and time, provides optional real-time subject feedback, or can be completely disabled.

Wear time sensor

Automatically detects if a wrist worn device has been removed for simplified compliance monitoring and data cleaning.

Inertial Measurement Unit (IMU)

Contains a secondary accelerometer and gyroscope and magnetometer sensors to capture position and rotation data for advanced applications.

Specifications

Dimensions	3.5 x 3.5 x 1 cm	Dynamic range (primary accelerometer)	+/- 8G	Water resistance	1 meter, 30 min.
Weight	14 grams	Dynamic range (secondary accelerometer)	+/- 16G	Wear location	Wrist, waist, ankle, thigh
Sample rate	30-100 Hertz	Gyroscope dynamic range	+/- 2000 deg/sec	Warranty	1 year
Battery life	14 days*	Magnetometer dynamic range	+/- 4800 micro-Tesla		
Data storage	180 days/4 GB	Communication	USB, Bluetooth® LE		

* Rechargeable Lithium Ion, wireless and gyro disabled, 30 Hz sample rate

This User Guide provides instructions on how to setup, deploy, and download data from the ActiGraph Link with the ActiLife software.

What You'll Need

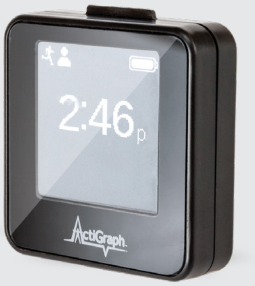

ActiGraph Link

ActiLife software (version 6.11.5 or higher)

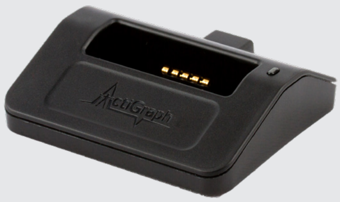

Single Dock

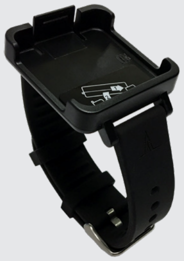

Link Wrist Band

Or

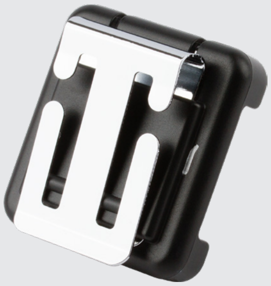

Link Belt Clip

Or

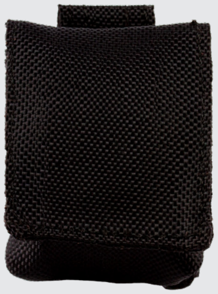

Pouch with Belt Loop

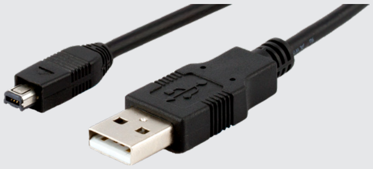

USB Cable(s)

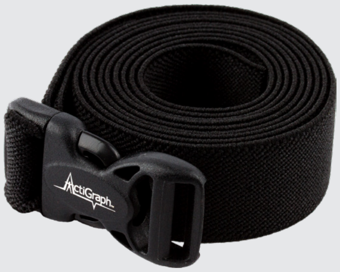

Waist Belt (Optional)

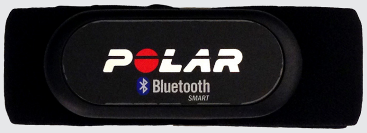

Heart Rate Monitor (Optional)

1

Install ActiLife software

You must be running ActiLife version 6.11.5 or higher to operate the ActiGraph Link device.

1. Go to
   https://actigraphcorp.com/actilife
   and click the **'Download'** button. Follow the prompts to install the ActiLife software on your PC.

2. When prompted, enter the ActiLife license key that was provided at the time of purchase to complete the installation.

View ActiLife System Requirements

Note: The ActiLife full version and ActiLife Lite can both be used to initialize and download data from the ActiGraph Link. However, the ActiLife full version is required to view and/or process the collected data.

2

Charge the battery

ActiGraph Link devices contain a rechargeable battery that should be fully charged before initialization and deployment to subjects. ActiLife will not initialize a device if the battery has dropped below a certain level. It takes approximately 2-3 hours to charge a fully depleted battery.

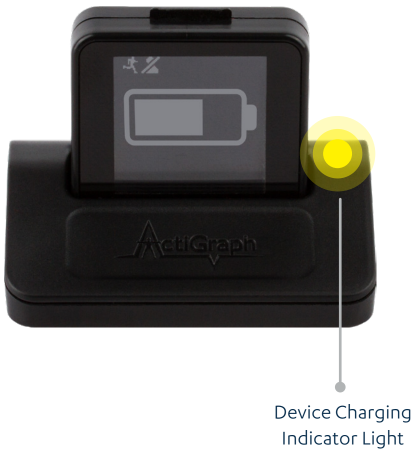

1. Connect the Link dock to the computer or a wall outlet using the mini USB cable.

2. Plug the ActiGraph Link into the dock with the ActiGraph logo facing up. Once connected, the red LED light on the right side of the dock will turn yellow, the device screen will display the serial number, and the battery icon will blink to indicate charging.

3. Once the device is fully charged, the yellow light will turn green and the battery icon on the device will show as full and stay on steady.

Charging multiple devices

To charge multiple ActiGraph Link devices simultaneously, plug up to six devices into the six port Link dock and connect to the PC and a wall power outlet using the supplied cables. The corresponding indicator light on the dock will turn green once each device is fully charged.

Note: ActiLife software is not required for battery charging.

Note: Battery voltage can be checked by opening the ActiLife software, connecting the device, and viewing the 'Battery' column in the grid under the 'Devices' tab. Select 'Refresh' to update the voltage level during charging.

Battery
3.44V (5% Normal)

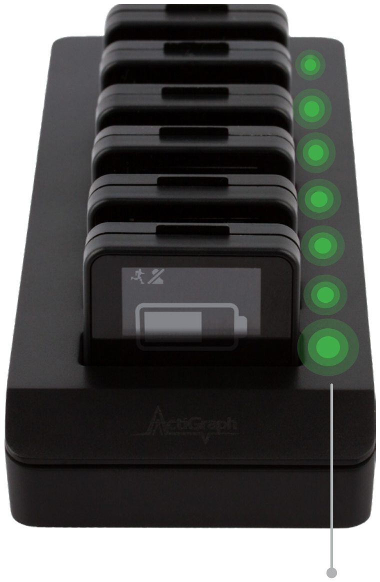

Device Charging Indicator Light

Battery life specifications

The ActiGraph Link has several features that impact battery life when enabled. Estimated battery life for common device configurations are as follows:

Display On, Wireless/Heart Rate Off, IMU Off: 14 days
Display On, Heart Rate On, IMU Off: 7 days
Display On, Wireless/Heart Rate Off, IMU On (all sensors): 1 day

* Default sample rate of 30 Hz, raw data collection mode, idle sleep modes

Note: These estimates are based on average device usage parameters. More frequent use of wireless communication will result in reduced battery life.

1. Open the ActiLife software.

2. Connect the ActiGraph Link to the PC using the Link dock. The monitor will appear in the grid under the **'Devices'** tab.

3. Select **'Initialize.'** A submenu will open to display several initialization options.

4. Select **'Regular Initialization'** from the submenu. A dialog box will open to display the initialization parameters listed below. The most commonly used initialization parameters are pre-selected as system defaults.

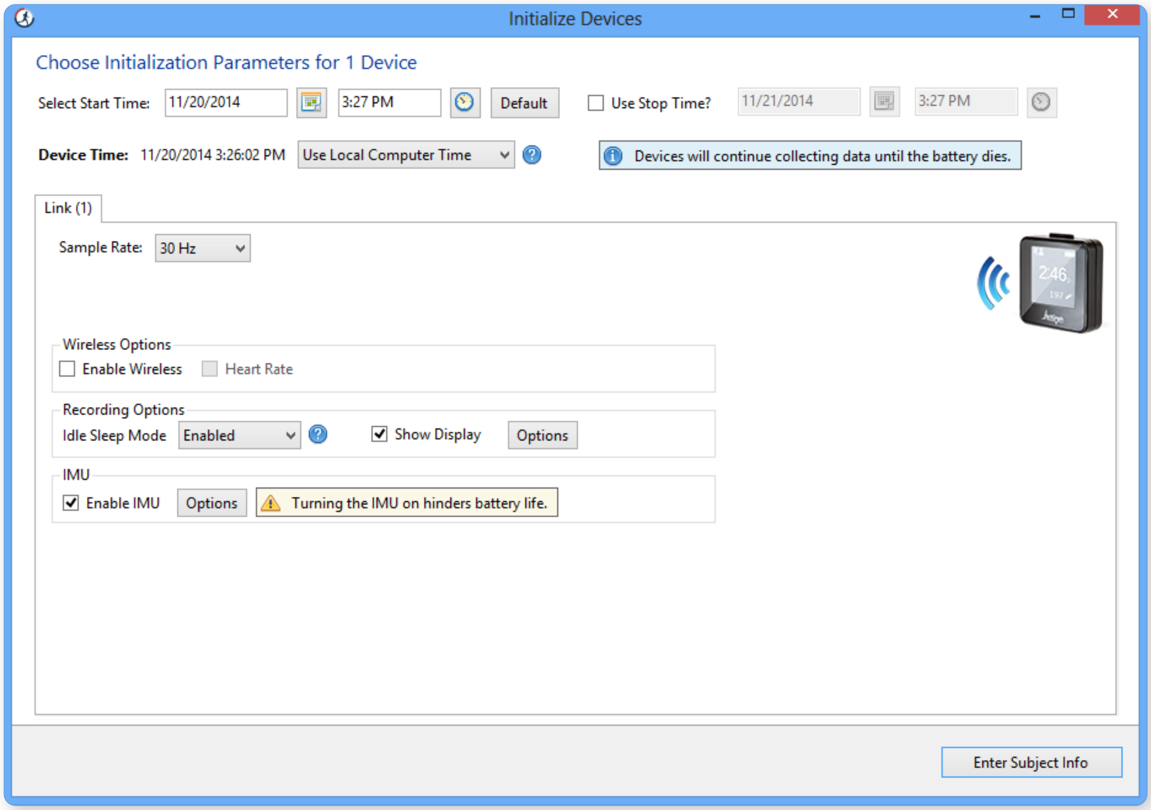

- ## Start and Stop Times
  Enter the dates and times when the device should start and stop collecting data. If no start time is selected, the system defaults to two minutes ahead. If no stop time is selected, the device will continue to collect data until the battery is depleted, the memory capacity is full, or it is downloaded and reinitialized.

- ## Device Time
  Set device to local computer time or atomic time.

- ## Sample Rate
  Select the device sampling rate ranging from 30 to 100 Hz. Note that higher sampling rates will result in reduced device storage capacity and battery life.

- Wireless Options
  - **Enable Wireless**
    Activates Bluetooth® functionality.
  - **Heart Rate**
    Capture heart rate data when device is used with compatible Bluetooth® heart rate monitor. Note that wireless must be enabled to activate heart rate data collection.

    Note: Activating the heart rate option automatically disables mobile app communication.
- Recording Options
  - **Idle Sleep Mode**
    When enabled, the device enters a low power state after experiencing 10 seconds of inactivity in order to preserve battery life.
  - **Show Display**
    Activates the display window on the device. Click **'Options'** to select whether to display date and time in 24 hour format and to enable subject feedback.
- IMU
  Enables Inertial Measurement Unit (IMU), which contains a gyroscope, magnetometer, secondary accelerometer, and IMU temperature sensor. The IMU data are collected at a 100 Hz sample rate.*

  Note: Enabling the IMU will result in significantly reduced device battery life and data storage capacity.

5. Complete the initialization parameters form and select **'Enter Subject Info.'**

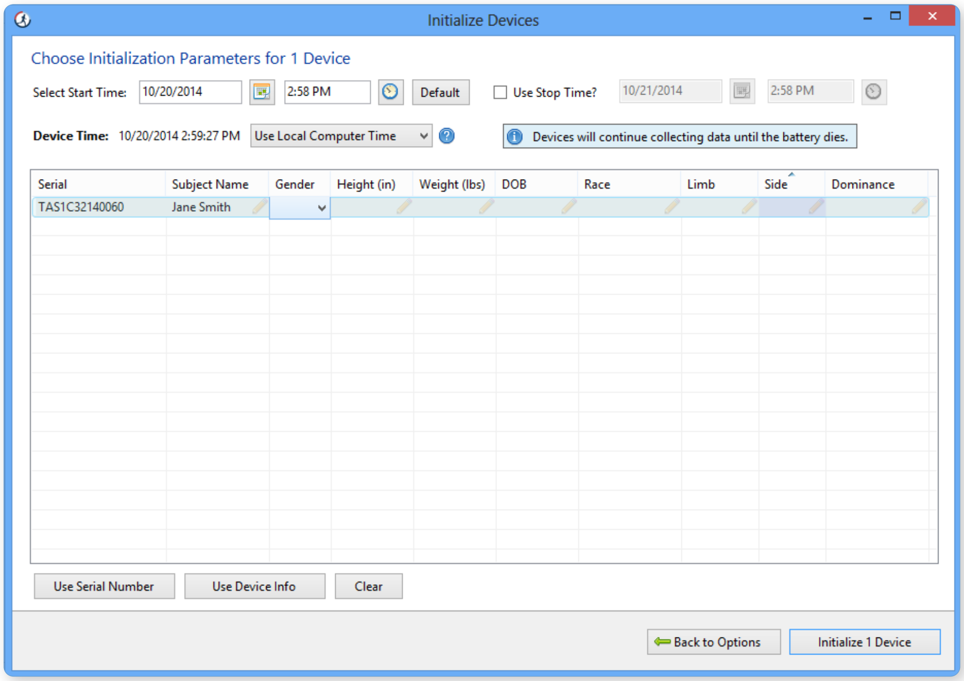

* ActiGraph products are not intended to diagnose, treat, cure, or prevent any disease.

6. Enter subject name, biometric information, and wear position details into the grid. Note that a subject name is required for initialization, but other biometric and wear details can be entered during download or data analysis.

7. Select **'Initialize 1 Device.'** A progress bar in the devices grid will indicate when the initialization process is completed.

8. After initialization is complete, remove the ActiGraph Link from the dock. Once removed, a 10 second wear sensor calibration countdown will begin. For wrist worn devices, immediately insert device into watch band (see Wearing the ActiGraph Link below) and lay it down on a flat surface until the countdown is complete. This calibration procedure is not required for waist worn devices, which do not provide valid wear sensor data.

9. The device will begin collecting data when the selected start time elapses, as indicated by the active mode icon in the upper left corner of the display. The device will continue to collect data until the stop time occurs (if selected), the battery is depleted, or the device is downloaded and reinitialized.

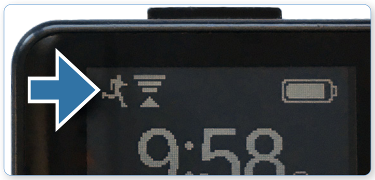

Note: If the start time elapses before the initialization process is complete, an error message will appear. Simply select the 'Default' button in the Start Time section to reset the default start time and then select 'Initialize 1 Device.'

Note: Multiple devices can be initialized simultaneously using the six port Link dock and/or multiple single Link docks. In this case, all devices will be programmed with the same initialization parameters and the Subject Info grid will display serial numbers of all connected devices for assignment.

1

ActiGraph Link Screen Icons

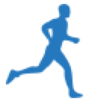

Active Mode
Device is collecting data.

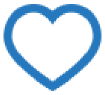

Heart Rate Not Connected
Device is configured to collect heart rate, but is not connected to a heart rate monitor.

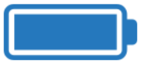

Battery
Battery level in 20% increments. Icon cycles during charging.

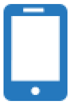

Mobile Connect
Device is communicating with mobile app via Bluetooth®.

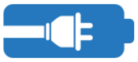

Battery Warn
Battery has reached critically low level. Estimated remaining charge (%) is displayed.

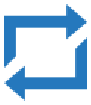

Reset Mode
Device is in a low power state.

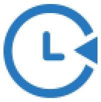

Delay Mode
Device has been initialized but start time has not yet elapsed.

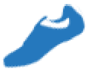

Steps
Shows steps accumulated for current day (12:00:00am - 11:59:59pm).

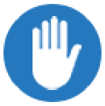

Halt
Device is in halt mode and not collecting data. Either the stop time has elapsed or the battery was fully depleted and recharged.

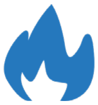

Calorie Expenditure
Shows calories burned for current day (12:00:00am - 11:59:59pm).

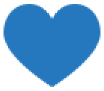

Heart Rate Connected
Device is collecting HR data. Beats per minute (BPM) will appear if display is enabled.

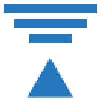

Wireless Broadcaster
Wireless is enabled.

2

Wearing the ActiGraph Link

The ActiGraph Link will typically be worn at the waist or on the non-dominant wrist. The appropriate wear location is dependent on specific research objectives and will be outlined in the study protocol.

Note: The device must be worn at the wrist location to obtain accurate readings from the wear time sensor.

Note: The device must be worn at the wrist location to obtain accurate sleep score information.

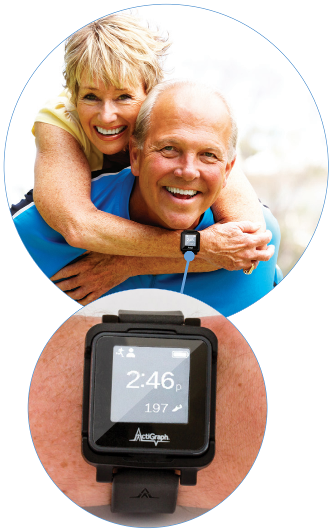

Wrist worn devices

Insert the ActiGraph Link into the wrist strap by positioning the plastic notch on the bottom edge of the device into the matching groove in the bottom edge of the watchband. Ensure that the ActiGraph logo on the device and the **'A'** on the watchband are facing in the same direction. Gently push down on the upper portion of the device until it snaps into place.

The subject should be instructed to wear the device strapped securely to the non-dominant wrist with the logo facing up when viewed like a wrist watch.

To remove the ActiGraph Link from the watchband, firmly grasp and pull up on the device with one hand while gently lifting the plastic tab on the top edge of the watchband with the other hand.

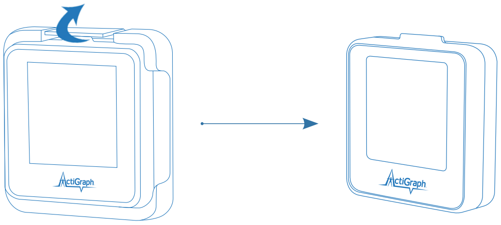

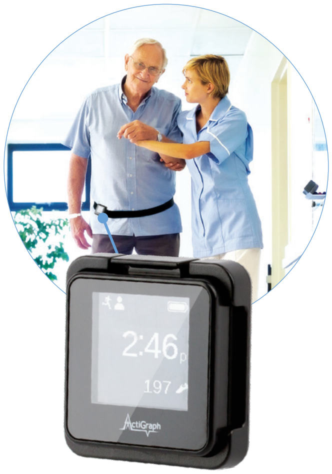

Waist worn devices

Belt Clip

Insert the ActiGraph Link into the belt clip by positioning the plastic notch on the bottom edge of the device into the matching groove on the bottom edge of the belt clip. Ensure that the ActiGraph logo faces up when the clip opening faces down. Clip the device to the elastic waist belt or the subjects' own belt or waistband. The belt should be fastened securely against the subject so the device is snug against the body.

To remove the ActiGraph Link from the belt clip, firmly grasp and pull up on the device with one hand while gently lifting the plastic tab on the top edge of the belt clip with the other hand.

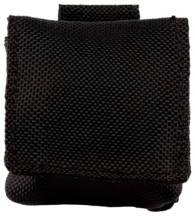

Pouch with Belt

Insert the ActiGraph Link into the belt pouch and secure flap using velcro tab. Thread an elastic waist belt or the subject's own belt through the loop on the back of the pouch. The belt should be fastened securely against the subject so the device is snug against the body.

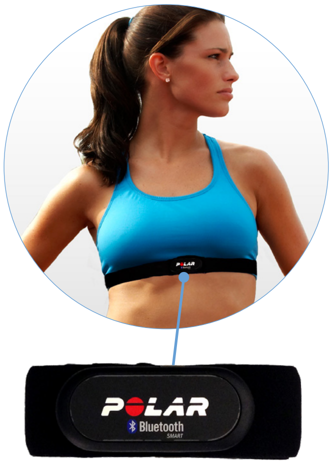

Heart Rate Monitor

To collect heart rate information, the **'Heart Rate'** option must be enabled during initialization and a compatible Bluetooth® wireless heart rate monitor is required. Unsnap the heart rate transmitter from the chest strap, position the transmitter in the center of the sternum, and snap back into place. Adjust the strap so that it is secure across the breast bone.

The ActiGraph Link will automatically begin collecting heart rate information when in range of the chest strap, as indicated by the heart rate screen icon on the device.

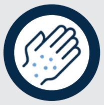

Wear the activity monitor only with the ActiGraph-approved wristband or belt clip provided. Failure to do so could result in device malfunction and possible skin irritation caused by direct contact with the conductive materials on the back of the device.

1. Open the ActiLife software.
2. Connect the ActiGraph Link to the PC using the dock station. The monitor will appear in the grid under the **'Devices'** tab.
3. Verify the box in front of the device is checked and select **'Download'** from the taskbar menu.
4. A dialog box will open to display the download options listed below. The most commonly used download parameters are preselected as defaults.

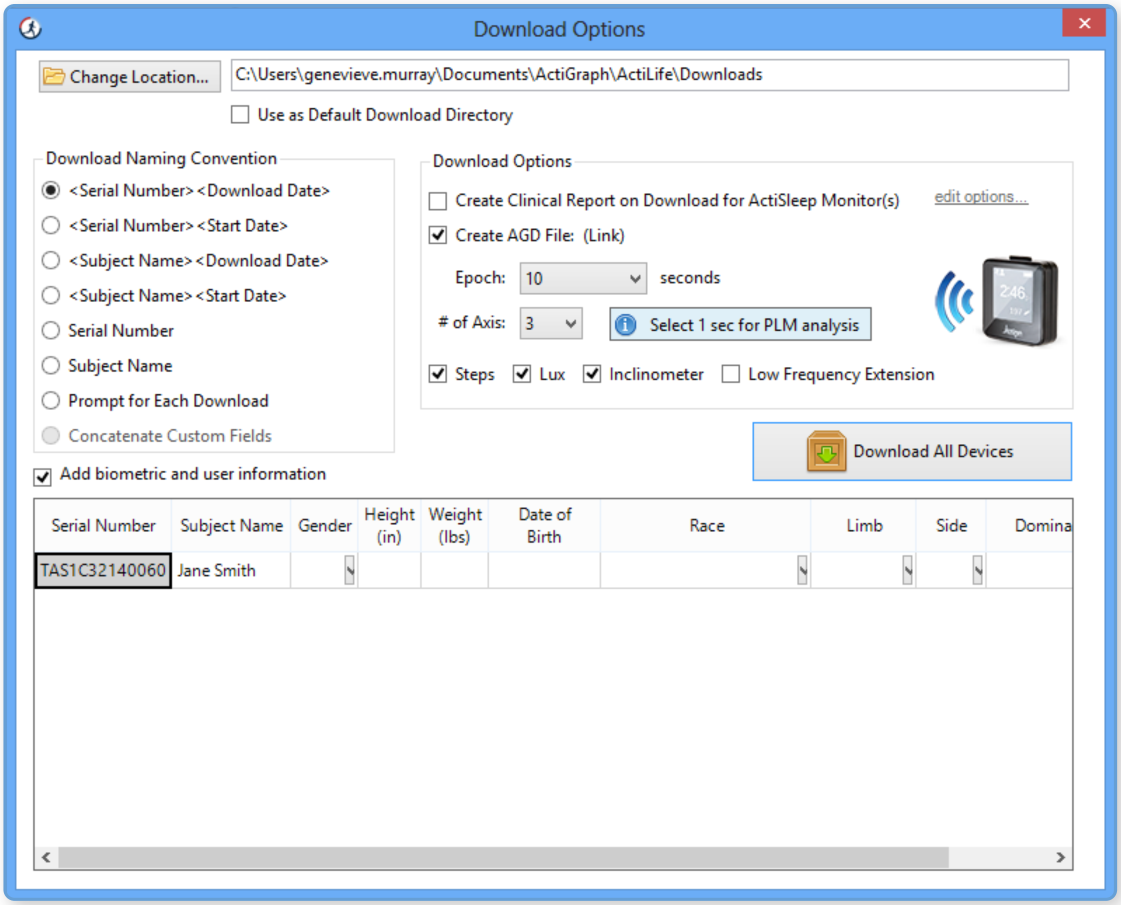

b File download location

Select where downloaded files will be saved.

c Download naming convention

Provides a list of file name formats.

Download Options:

- **Create Clinical Report**
  A customizable PDF report containing summary data will be automatically generated on download. Subject measures and scoring algorithms can be selected by clicking **'edit options.'**

- **Create AGD File**
  An AGD file, required for data scoring, will be automatically created on download.
  - **Epoch:** Select the desired epoch length for the AGD file.
  - **# of Axis:** Select which axes of data should be included in AGD file.

    Note: Axis 1=Y; Axis 2=X,Y; Axis 3=X,Y,Z
  - **Steps:** Select to include step count data in AGD file.
  - **Inclinometer:** Select to include positional data in AGD file.
  - **Low Frequency Extension:** Select to apply low frequency filter extension to AGD file.

    Note: The Low Frequency Extension option should only be used in very specific use cases where physical activity is at such a low level that it might otherwise be eliminated with our normal filter. An example would be very slow shuffling movements, common in elderly populations.

d. **Biometric and User Information**
Subject biometric information can be entered and/or edited. Fields will be pre-populated if this information was entered during initialization.

5. Once Download Options are selected, click **'Download All Devices.'** The dialog box will close and a progress bar will appear under the **'Status'** column in the grid.

6. When the download is complete, a **'finished downloading'** link will appear. Click this link to reveal options to view data, export the raw data file, and navigate to the download folder.

7. Data has now successfully been downloaded from the ActiGraph Link and can be cleaned and scored using the analysis tools in ActiLife.

Note: Collected data will remain on the ActiGraph Link device until it is reinitialized.

Viewing the IMU Data

1. After completing the download, select **File** > **Import/Export/Convert** > **IMU** in the ActiLife task bar.
2. Select the raw (.gt3x) file from the list and click **'Open.'**
3. A status bar will appear while ActiLife converts the raw file to CSV. Once completed, the file location window will open and the IMU file will appear in the list.

About the IMU file

The IMU file will contain some or all of the following information based on the IMU selections made during initialization. The sample rate of the IMU file is fixed at 100 Hz.

Timestamp	Accelerome	Acceleromet	Accelerometer	Temperature	Gyroscope X	Gyroscope Y	Gyroscope Z	Magnetometer X	Magnetomete	Magnetom
2014-11-04T09:25:59.0000000	0.418945	-0.60791	0.327637	63.016354	69.763188	-28.564455	13.977052	27.246092	9.667968	15.234374
2014-11-04T09:25:59.0100000	0.441406	-0.554688	0.32666	63.726211	51.635745	-48.828128	9.216309	27.246092	9.667968	15.234374
2014-11-04T09:25:59.0200000	0.475586	-0.530273	0.333496	63.78312	34.790041	-74.157719	3.90625	27.246092	9.667968	15.234374
2014-11-04T09:25:59.0300000	0.48584	-0.477539	0.293945	63.768144	31.127932	-94.421392	-2.624512	27.246092	9.667968	15.234374
2014-11-04T09:25:59.0400000	0.439453	-0.373535	0.26709	63.768144	33.447268	-97.10694	-11.413575	27.246092	9.667968	15.234374
2014-11-04T09:25:59.0500000	0.289551	-0.293945	0.527344	63.759158	49.56055	-54.382328	-16.967774	27.246092	9.667968	15.234374
2014-11-04T09:25:59.0600000	0.225098	-0.227539	0.425781	63.753168	83.190923	-15.502931	-2.685547	27.246092	9.667968	15.234374
2014-11-04T09:25:59.0700000	0.270996	-0.169922	0.38623	63.768144	102.050787	-10.864258	21.240236	27.246092	9.667968	15.234374
2014-11-04T09:25:59.0800000	0.330566	-0.135254	0.400879	63.759158	114.807136	1.831055	39.489749	27.246092	9.667968	15.234374

Timestamp

- The timestamps shown in the exported .csv file are formatted to show the full date in YYYY-MM-DD format. The "T" symbol indicates the beginning of the time element for the timestamp. The time is formatted as hh:mm:ss.ss where the last "ss" represents the fractional portion of the timestamp. Each entry increments by 0.01 seconds (because of the 100Hz fixed sample rate)

Accelerometer X, Y, Z

- Each accelerometer entry represents instantaneous acceleration for the axis indicated in units of gravity (Gs). Device orientation is shown in this help article.

- The accelerometer on the IMU has a dynamic range of +/- 16Gs per axis

Gyroscope X, Y, Z

- Gyroscope measurements from the IMU represent are presented in degrees/sec for each axis. Refer to this help article for details regarding roll/pitch/yaw orientation.

Magnetometer X, Y, Z

- The IMU magnetometer readings represent the magnetic field experienced by the Link device and are useful for discerning directional orientation (compass). These readings are in microTesla (µT)

IMU Temperature

- The IMU temperature reading indicates ambient temperature inside of the Link activity monitor and is indicated in degrees Celsius (C).

Note: The accelerometer data in the IMU file is not from the primary accelerometer and is not used for scoring in ActiLife.

1

Regulatory statements

This device complies with Part 15 of the FCC Rules. Operation is subject to the following two conditions: (1) This device may not cause harmful interference, and (2) this device must accept any interference received including interference that may cause undesired operation. Changes or modifications not expressly approved by ActiGraph, LLC will void the user's authority to operate the equipment under FCC regulations.

- *FCC Part 15.107 – AC Conducted Emissions*
- *FCC Part 15.109 – Radiated Emissions*
- *FCC Part 15.207 – Modular Transmitter AC Line Conducted Emissions*
- *FCC Part 15.249 – Radiated Emission Limits of Intentional Radiators*

Note: This equipment has been tested and found to comply with the limits for a Class B digital device, pursuant to part 15 of the FCC Rules. These limits are designed to provide reasonable protection against harmful interference in a residential installation. This equipment generates, uses and can radiate radio frequency energy and, if not installed and used in accordance with the instructions, may cause harmful interference to radio communications. However, there is no guarantee that interference will not occur in a particular installation. If this equipment does cause harmful interference to radio or television reception, which can be determined by turning the equipment off and on, the user is encouraged to try to correct the interference by one or more of the following measures:

- *Reorient or relocate the receiving antenna*
- *Increase the separation between the equipment and receiver*
- *Connect the equipment into an outlet on a circuit different from that to which the receiver is connected*
- *Consult the dealer or an experienced radio/TV technician for help*

2

Regulatory symbols

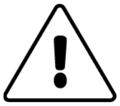

CAUTION: Do not simultaneously wear and charge, service, or provide any maintenance on the product.

CAUTION: Transporting or operating this product outside of the temperature range of -20C to 40C could lead to dangerous conditions.

CAUTION: Modification to ActiGraph products are not permitted and will void all warranties if tampered and/or modified. Do not modify the product in any way.

CAUTION: Do not swallow any part of this product. If a piece has been swallowed, contact your local poison control hotline or seek medical attention as soon as possible.

NOTICE: No precautions need to be taken in the event of changes in the performance of the product.

NOTICE: Product does not have any contraindication(s)

NOTICE: Only use a USB 2.0 cable to connect to the device. Only use ActiGraph approved USB hubs to charge the device. Do not connect the device via the USB 2.0 port to anything else but a computer and ActiGraph approved USB hub.

EC REP

Emergo Europe
Prinsessegracht 20
2514 AP The Hague
The Netherlands

Authorized Representative in European Community

Indicates the authorized representative in the European Community.

AUSTRALIAN SPONSOR
Emergo Australia
Level 20, Tower II
Darling Park
201 Sussex Street
Sydney NSW 2000
Australia

ActiGraph's Australia Sponsor

Indicates ActiGraph's authorized Australian Sponsor.

REF

Catalog Number

Indicates the manufacturer's catalogue number so that the medical device can be identified for reordering.

CE

CE Symbol

By affixing the CE marking to a product, a manufacturer declares that the product meets all the legal requirements for CE marking and can be sold throughout the EEA. ActiGraph's products abide with the Medical Device Directive 93/42/EEC and Radio Equipment Directive 2014/53/EU.

ANATEL
06587-19-09607
"Este equipamento não tem direito à proteção contra interferência prejudicial e não pode causar interferência em sistemas devidamente autorizados."

Brazil National Telecommunications Agency (ANATEL) Homologação Number.

This equipment operates on a secondary basis, that is, not entitled to protection from harmful interference, even for stations of the same type, and may not cause interference to systems operating on a primary basis.

Consult Instructions for Use

Indicates the need for the user to consult the instructions for use.

SN

Serial Number

Indicates the manufacturer's serial number so that a specific medical device can be identified.

Manufacturer

ActiGraph is the medical device manufacturer and is located at 49 E. Chase Street, Pensacola, Florida 32502.

-20C 40C

Temperature Limit

Indicates the temperature limits to which the medical device can be safely exposed.

Recycle: Electronic Equipment

Indicates the medical device should not be disposed of in the trash. Contact ActiGraph Customer Service regarding the disposal of these products.

Li-ion

Lithium Ion (EU)

Indicates the lithium ion battery within the device should not be disposed of in the trash. Contact ActiGraph Customer Service regarding the disposal of these batteries.

Q Is the ActiGraph Link waterproof?

The ActiGraph Link is water resistant to 1 meter for 30 minutes according to IP27 certification. The device can be worn during bathing and swimming activities.

Q How should I clean the ActiGraph Link and accessories after use by a subject?

The ActiGraph Link, wristband, and belt clip should be wiped down using any alcohol based solution. Elastic belts and pouches should be laundered.

Q Is the raw accelerometer output the same as with ActiGraph's previous devices?

Yes, the ActiGraph Link uses the same accelerometer and sampling and filtering methods as the GT3X+ line of activity monitors.

Q Is there any way for the subject to change or reprogram the ActiGraph Link display after it has been deployed?

No, the ActiGraph Link display will operate according to the parameters selected during initialization until it has been reinitialized with ActiLife.

Q Does the wear sensor work at the waist?

No, the wear sensor is only accurate for wrist worn devices.

Q What do the various sensors in the IMU measure?

The accelerometer measures acceleration normalized to Earth gravity (g). The gyroscope measures angular rates in degrees per second. The magnetometer measure magnetic field strength in microteslas. The thermometer measures temperature of the IMU in Celsius.

Q Does the magnetometer provide accurate measurements in any geographic location?

Variations in magnetic north versus true north vary by location in a predictable way. There are lookup tables available. The magnetic field measurements may also be affected by hard and soft iron effects.

Q Can I calculate body temperature using the thermometer in the IMU?

No, the thermometer measures the temperature of the the IMU sensor for possible compensation of the IMU output.

Q What do I do in case of an allergic reaction?

Report to your study site team any known allergies to steel or rubber prior to wearing the activity monitor.
If the device becomes uncomfortable to wear, hot, or causes skin irritation, remove it and contact the study site team immediately. Do not wear the activity monitor over any open wounds or irritated skin.

ActiGraphCorp.com

Please contact our Customer Support Team with any questions or for additional information about operating the ActiGraph GT9X Link.

49 East Chase Street. Pensacola, FL 32502
support@actigraphcorp.com | 850.332.7900 | +1 (877) 497-6996 (toll free)

©2019 ActiGraph, LLC. All rights reserved.

U.S. federal law restricts this device to sale by or on the order of a physician.
